# LY2444296, a κ-opioid receptor antagonist, selectively reduces alcohol drinking in male and female Wistar rats with a history of alcohol dependence

**DOI:** 10.1038/s41598-024-56500-9

**Published:** 2024-03-09

**Authors:** Francisco J. Flores-Ramirez, Jessica M. Illenberger, Glenn Pascasio, Lars Terenius, Rémi Martin-Fardon

**Affiliations:** 1 Department of Molecular Medicine, The Scripps Research Institute, 10550 North Torrey Pines Road, SR-107, La Jolla, CA 92037 USA; 2 Department of Clinical Neuroscience, Karolinska Institute, Stockholm, Sweden

**Keywords:** Alcohol use disorder, Dynorphin, Kappa-opioid receptor antagonist, LY2444296, Neuroscience, Motivation

## Abstract

Alcohol use disorder (AUD) remains a major public health concern. The dynorphin (DYN)/κ-opioid receptor (KOP) system is involved in actions of alcohol, particularly its withdrawal-associated negative affective states. This study tested the ability of LY2444296, a selective, short-acting, KOP antagonist, to decrease alcohol self-administration in dependent male and female Wistar rats at 8 h abstinence. Animals were trained to orally self-administer 10% alcohol (30 min/day for 21 sessions) and were made dependent via chronic intermittent alcohol vapor exposure for 6 weeks or exposed to air (nondependent). After 6 weeks, the effect of LY2444296 (0, 3, and 10 mg/kg, p.o.) was tested on alcohol self-administration at 8 h of abstinence. A separate cohort of rats was prepared in parallel, and their somatic withdrawal signs and alcohol self-administration were measured after LY2444296 administration at 8 h, 2 weeks, and 4 weeks abstinence. LY2444296 at 3 and 10 mg/kg significantly reduced physical signs of withdrawal in dependent rats at 8 h abstinence, only. Furthermore, 3 and 10 mg/kg selectively decreased alcohol self-administration in dependent rats at only 8 h abstinence. These results highlight the DYN/KOP system in actions of alcohol during acute abstinence, suggesting KOP antagonism could be beneficial for mitigating acute withdrawal signs and, in turn, significantly reduce excessive alcohol consumption associated with AUD.

## Introduction

Alcohol use disorder (AUD) is among the most prevalent mental disorders and remains a major public health, economic, and social concern both in the United States and around the world^1–3^. One of its major hallmarks is the loss of control to reduce or stop drinking, which may lead to aggressive and harmful behavior such as driving under the influence, and repeated episodes of excessive drinking may lead to hepatitis, fibrosis, and cirrhosis of the liver^4^. Heavy drinking over extended periods of time injures the liver first and to the greatest extent and is also associated with other negative outcomes because it can damage nearly every other organ in the body^4,5^. Indeed, AUD can lead to different types of cancers and cardiovascular disease and is the seventh leading cause of disability and preventable cause of death worldwide and third leading cause in the United States^6,7^. With the goal of reducing drinking and achieving and maintaining long-term abstinence from alcohol use, several behavioral, psychosocial, and pharmacological approaches are available for those who suffer from AUD^8^. To date, three pharmacotherapies have been approved by the United States Food and Drug Administration (FDA) for the treatment of AUD: disulfiram, acamprosate, and naltrexone^8–10^. Unfortunately, although these medications have proven effective in reducing heavy drinking and prolonging abstinence, they are still underutilized by medical professionals clinically^11^. Therefore, more research is needed to characterize new pharmacological targets to develop alternative pharmacotherapies for AUD that are both safe and effective.

The dynorphin (DYN)/κ-opioid receptor (KOP) system has received interest in efforts to develop AUD-specific therapeutics, given its involvement in actions of alcohol, particularly negative affective states that are associated with withdrawal^12^. Encoded by the preprodynorphin (*Pdyn*) gene, DYN derives from the precursor prodynorphin (pDYN) and exists in different forms, including DYN A and DYN B, possessing high affinity for KOPs^12^. Notably, both the DYN peptide and its receptor are widely distributed throughout the brain, with strong expression in the medulla, pons, hippocampus, hypothalamus, thalamus, striatum, and cortex^13–15^. In line with the diverse functionality of these brain regions, DYN is thought to play a significant role in the regulation of different processes, including learning, memory, emotional regulation, stress, and pain and has been highlighted as a marker of interest in the pathophysiological mechanisms that underlie epilepsy, depression, schizophrenia, chronic pain, and particularly reward mechanisms and drug dependence^15^. Several studies have described a high density of KOPs in mesolimbic dopamine pathway neurons and provided evidence that DYN plays a regulatory role in dopaminergic tone, thereby mediating reward, reinforcement, aversion, and dysphoria^16–18^.

The DYN system throughout the brain, particularly KOP signaling, is well known to be affected by both acute and chronic exposure to alcohol. Notably, a previous study showed that three intragastric administrations of 1.5 g/kg alcohol (in a single day) were sufficient to upregulate the expression of *Pdyn* in the amygdala and prefrontal cortex^19^, and it significantly increased DYN levels in the nucleus accumbens (NAC), ventral tegmental area (VTA), central nucleus of the amygdala (CeA), and paraventricular nucleus of the thalamus (PVT)^20–23^. Similarly, DYN was upregulated in the NAC and periaqueductal gray in rats 30 min after 13 days of intraperitoneal alcohol administration (2 g/kg twice daily), an effect that remained even 21 days later^24^. Similarly, some studies that used chronic intermittent ethanol (CIE) vapor exposure found an increase in KOP function 72 h after the last exposure to alcohol^25,26^. In rats, during acute withdrawal after alcohol dependence induction with CIE vapor exposure, a significant upregulation of KOP gene (*Oprk1*) expression was observed in the bed nucleus of the stria terminalis (BNST)^27^.

Furthermore, the pharmacological activation of KOPs with a receptor agonist increased alcohol-seeking behavior and the likelihood of exacerbated drinking^28–30^, and these effects were accompanied by an increase in anxiety- and depression-like behavior that may, at least, partially underlie uncontrolled drinking^16,31^. Several studies have also shown that the manipulation of DYN transmission with a KOP antagonist regulates alcohol intake. For example, the escalation of drinking that is induced by CIE vapor exposure was reversed by the systemic blockade of KOPs in mice^26,32^. Similarly, the administration of norbinaltorphimine (norBNI) significantly decreased alcohol intake in Wistar rats that were made dependent by CIE vapor exposure but not in their nondependent counterparts^33^. Additionally, administration of the KOP antagonist CERC-501 (also known as LY2456302) reduced alcohol self-administration in alcohol-preferring rats, and this attenuation of alcohol intake was accompanied by a reduction of depression-like behavior^34^. Following long-term intermittent access to alcohol, LY2456302 ameliorated escalated drinking in rats^35^.

While efficacious, some of these antagonists that have been tested preclinically such as norBNI and (3R)-7-hydroxy-N-[(2S)-1-[(3R,4R)-4-(3-hydroxyphenyl)-3,4-dimethylpiperidin-1-yl]-3-methylbutan-2-yl]-1,2,3,4-tetrahydroisoquinoline-3-carboxamide (JDTic) display unusual pharmacokinetic and pharmacodynamic properties, delayed development of KOP selectivity, long-lasting effects, and poor solubilities, which hinders their potential for interpretation and translation into the clinic as possible therapeutic approaches. Indeed, both norBNI and JDTic have an onset of maximal KOP antagonist actions within 24–48 h, and these actions may last more than two weeks after a single administration^36,37^. To enhance our understanding of the DYN/KOP system involvement in the neurobiology of substance use disorders, several compounds with pharmacologically relevant durations of action have been synthesized. For example, ((S)3-fluoro-4-(4((2(3-fluo-rophenyl)pyrrolidin-1-yl)methyl)phenoxy)benzamide (LY2444296), is a highly selective short-acting KOP antagonist (Ki = 0.565 nM for KOP vs. 35.8 nM and 211 nM for mu and delta opioid receptors respectively; see^38^). LY244426’s selectivity for the KOP in vivo was demonstrated by its lack of effect in morphine antinociception in a formalin assay in rats, and by its potent antagonist activity onset occurring within 30 min oral dosing (compound 25 in^38^). Furthermore, LY2444296 significantly decreased escalated cocaine consumption, anxiety and depressive-like syndromes, as well as serum corticosterone levels in a rat model of extended access to cocaine self-administration^39,40^.

**Figure 1:**
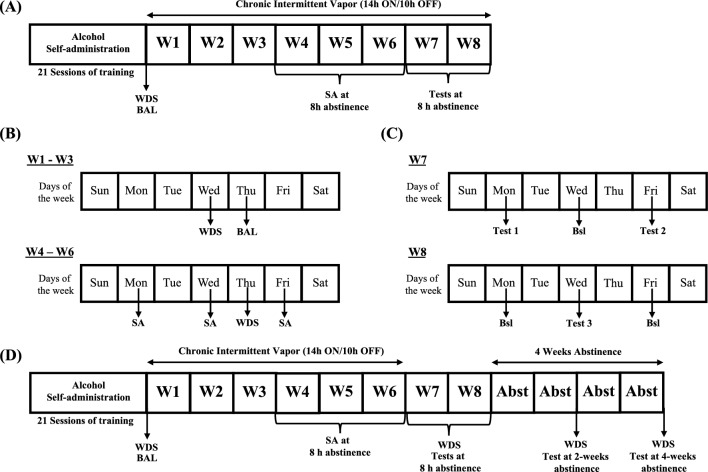
Timeline of the experimental procedures. (**A**) Male Wistar rats underwent 21 sessions of alcohol self-administration training. Upon the completion of training, baseline somatic withdrawal signs, alcohol intake, and BALs after self-administration were recorded. (**B**) The rats were scored for somatic withdrawal signs during acute abstinence (8 h after the vapor was turned OFF, on Wednesday), and BALs were recorded 30 min before the alcohol vapors were turned OFF (on Thursdays) between weeks 1 and 3 of CIE vapor exposure. The rats underwent self-administration sessions three times weekly (Monday, Wednesday, and Friday) during acute abstinence (8 h after alcohol vapor was turned OFF) between weeks 4 and 6 of CIE vapor exposure. (**C**) Between weeks 7 and 8, one group of rats was tested with LY2444296 (0, 3, and 10 mg/kg) at 8 h of abstinence in random order using a Latin-square design every other session. On days between testing, the rats underwent regular self-administration sessions, without LY2444296. (**D**) Effects of LY2444296 on somatic withdrawal signs and alcohol self-administration at 8 h, 2 weeks, and 4 weeks of abstinence. Between weeks 7 and 8, rats were tested with all doses of LY2444296 (0, 3, and 10 mg/kg) at 8 h of abstinence in random order using a Latin-square design every other session. At 2 and 4 weeks of abstinence, only the 0 and 10 mg/kg doses were tested. On days between testing, the rats underwent regular self-administration sessions without LY2444295. BAL, blood alcohol level; Bsl, baseline; SA, self-administration; WDS, somatic withdrawal signs; W, week; Abst, abstinence.

In summary, the extant literature that implicates the DYN/KOP system as a major player in the mechanisms that underlie AUD shows that exposure to alcohol, either acutely or chronically, upregulates activity of the DYN/KOP system throughout distinct brain regions and that manipulating KOP signaling via pharmacological antagonists may reduce drinking in postdependent subjects. Considering that a main characteristic of AUD is the loss of control over alcohol consumption that is directly related to avoiding negative affect during alcohol withdrawal^41^, the present study had two main goals: (1) test the ability of the newly developed pharmacological tool, LY2444296, to decrease alcohol self-administration at different abstinence points (acute [8 h], late [2 weeks], and protracted [4 weeks]) and (2) assess whether LY2444296 administration may also result in a parallel reduction of somatic withdrawal signs in male and female Wistar rats (Fig. 1).

## Results

Given that sex did not significantly contribute to the variance of the model in any of the dependent variables measured, data obtained from both male and female rats were analyzed together.

### Alcohol self-administration training and escalation

**Figure 2:**
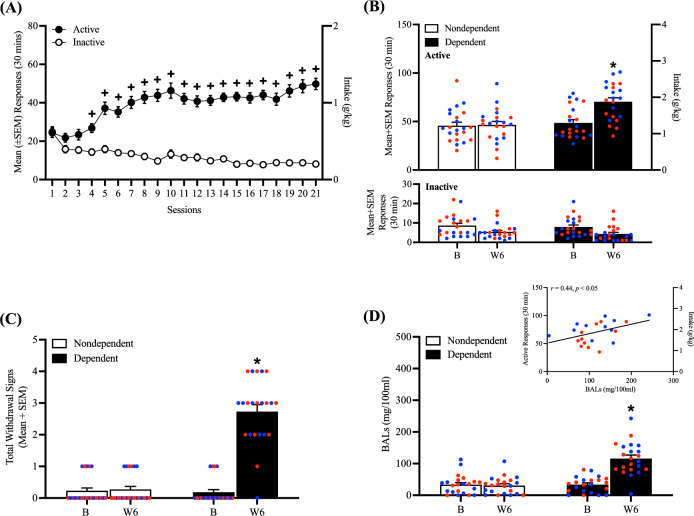
Time course of alcohol self-administration acquisition across 21 training sessions and escalation of drinking during week 6 of CIE vapor exposure. (**A**) Male and female rats acquired alcohol self-administration over the 21 training sessions. (**B**) At week 6 of CIE vapor exposure, alcohol-dependent rats exhibited a significant increase in alcohol self-administration. (**C**) A significant increase in somatic withdrawal signs was observed in dependent rats at week 6 of CIE vapor exposure during acute abstinence. (**D**) After the self-administration sessions at week 6 of CIE vapor exposure, alcohol-dependent rats exhibited significantly higher BALs. Alcohol self-administration and BALs at week 6 significantly correlated (inset). The data are expressed as the mean + SEM. ^+^*p* < 0.05, *vs.* inactive lever; *p < 0.05, versus respective baseline. Blue dots denote data from male rats. Red dots represent data from female rats. B, baseline; W, week.

Over 21 sessions of training (30 min/day), the rats acquired alcohol self-administration (mixed two-way analysis of variance [ANOVA]; time: *F*_20,1800_ = 4.39, *p* < 0.05; lever: *F*_1,90_ = 232.61, *p* < 0.05; time × lever interaction: *F*_20,1720_ = 17.75, *p* < 0.05; Fig. 2A. Tukey’s multiple-comparison post hoc test confirmed that the number of responses on the active lever were significantly higher than the number of responses on the inactive lever starting at the 4th session and for the remainder of self-administration training (*p* < 0.05).

During week 6 of CIE, alcohol-dependent rats exhibited an increase in alcohol self-administration, a measure that was obtained by averaging the number of responses on the active lever that were recorded Monday, Wednesday, and Friday of that week (*p* < 0.05, Bonferroni post hoc test vs. baseline following a mixed methods two-way ANOVA; time: *F*_1,42_ = 22.22, *p* < 0.05; alcohol dependence: *F*_1,42_ = 8.39, *p* < 0.05; time × alcohol dependence interaction: *F*_1,42_ = 19.29, *p* < 0.05; Fig. 2B). No differences in the number of responses on the inactive lever were observed, regardless of the rats’ histories of alcohol dependence (*p* < 0.05; Fig. 2B bottom panel).

During week 6 of CIE, alcohol-dependent rats exhibited significantly higher somatic withdrawal signs at an acute abstinence point (8 h after vapors were off; *p* < 0.05, Bonferroni post hoc test vs. baseline following a mixed methods two-way ANOVA; time: *F*_1,42_ = 109.17, *p* < 0.05; alcohol dependence: *F*_1,42_ = 68.20, *p* < 0.05; time × alcohol-dependence interaction: *F*_1,42_ = 101.64, *p* < 0.05; Fig. 2C).

During week 6 of CIE, alcohol-dependent rats had significantly higher blood alcohol levels (BALs) after the self-administration sessions of that week (*p* < 0.05, Bonferroni post hoc test *vs.* baseline following a mixed methods two-way ANOVA; time: *F*_1,42_ = 30.69, *p* < 0.05; alcohol dependence: *F*_1,42_ = 32.05, *p* < 0.05; time × alcohol-dependence interaction: *F*_1,42_ = 35.81, *p* < 0.05; Fig. 2D) that positively correlated with alcohol self-administration (*r*_20_ = 0.44, *p* < 0.05; Fig. 2D, inset).

### Effects of LY2444296 on alcohol self-administration

**Figure 3:**
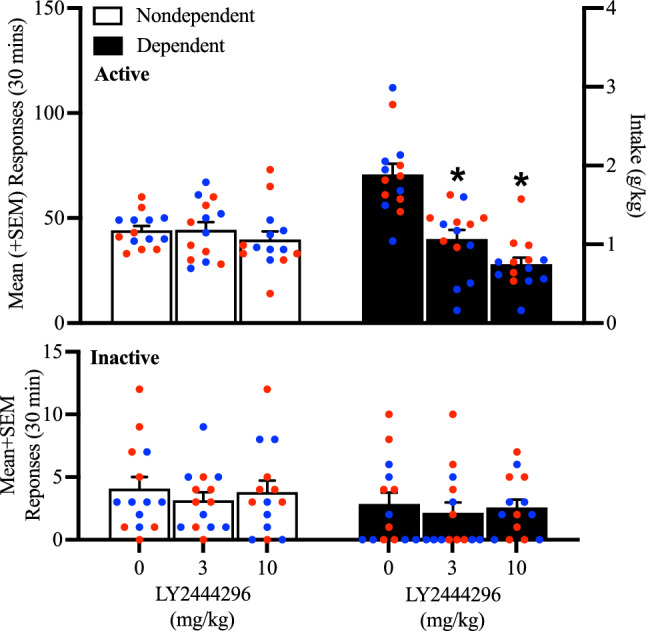
Effect of LY2444296 (0, 3, and 10 mg/kg) on alcohol self-administration at 8 h of abstinence. (**A**) The administration of LY2444296 significantly decreased alcohol self-administration in dependent rats at all doses (3 and 10 mg/kg) tested and did not produce any effects in nondependent rats. No differences in inactive lever responses were observed, regardless of treatment or alcohol dependence. The data are expressed as the mean + SEM. *p < 0.05, versus 0 mg/kg. Blue dots denote data from male rats. Red dots represent data from female rats.

After 6 weeks of CIE dependence induction, the ability of LY2444296 to attenuate alcohol self-administration was assessed. In nondependent male and female rats, LY2444296 administration, regardless of dose, did not affect alcohol self-administration when compared with vehicle (0 mg/kg). However, LY2444296 administration significantly decreased alcohol self-administration at the 3 and 10 mg/kg doses in alcohol-dependent rats (*p* < 0.05, Tukey post hoc test *vs.* vehicle following a mixed methods two-way ANOVA; dose: *F*_2,52_ = 24.57, *p* < 0.05; alcohol dependence × dose: *F*_2,52_ = 17.74, *p* < 0.05; Fig. 3 top panel). No differences in the number of responses on the inactive lever were observed, regardless of the rats’ histories of alcohol dependence or LY2444296 dose (*p* > 0.05; Fig. 3 bottom panel).

### Effects of LY2444296 on somatic withdrawal signs and alcohol self-administration

**Figure 4:**
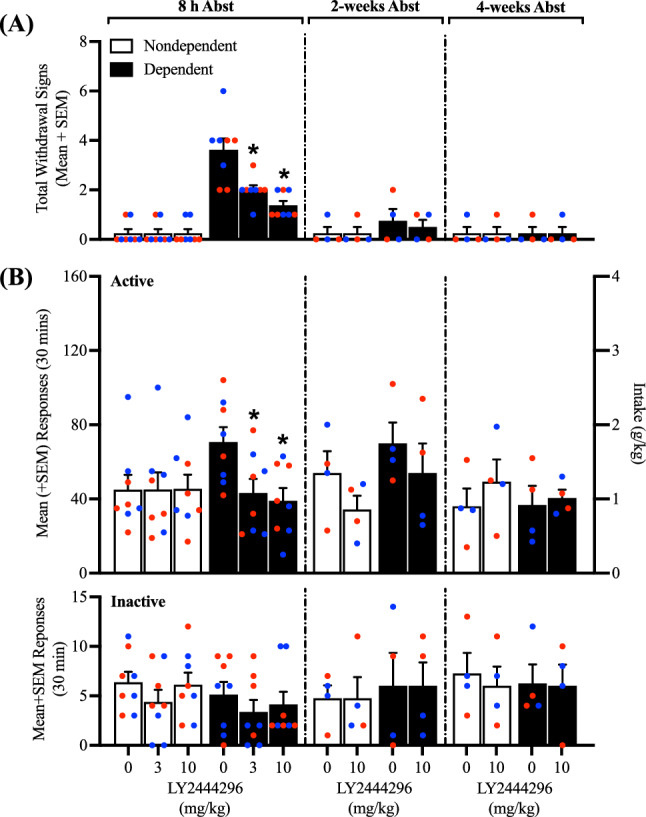
Effect of LY2444296 (0, 3, and 10 mg/kg) on somatic withdrawal signs and alcohol self-administration at 8 h of abstinence and 0 and 10 mg/kg at 2 and 4 weeks of abstinence. (**A**) The administration of LY2444296 significantly decreased somatic withdrawal signs in dependent rats at all doses (3 and 10 mg/kg) tested and did not produce any effects in nondependent rats at 8 h of abstinence. No differences in somatic withdrawal signs were observed at 2 or 4 weeks of abstinence. (**B**) The administration of LY2444296 at 8 h of abstinence significantly decreased alcohol self-administration in dependent rats at all doses (3 and 10 mg/kg) tested, without producing any effects in nondependent rats. No differences were observed at 2 or 4 weeks of abstinence, regardless of treatment or history of dependence. No differences in inactive lever responses were observed, regardless of treatment or alcohol dependence. The data are expressed as the mean + SEM. *p < 0.05, versus 0 mg/kg. Blue dots denote data from male rats. Red dots represent data from female rats.

At the end of week 6 of CIE vapor exposure, the ability of LY2444296 to attenuate somatic withdrawal signs and alcohol self-administration at 8 h, 2 weeks, and 4 weeks of abstinence was tested. Interestingly, alcohol-dependent male and female rats that received LY2444296 exhibited significantly lower somatic withdrawal signs at 8 h of abstinence at both the 3 mg/kg (*t*_14_ = 3.26, *p* < 0.05) and 10 mg/kg (*t*_14_ = 4.54, *p* < 0.05) doses in alcohol-dependent rats (Fig. 4A). In nondependent rats, LY2444296 administration did not affect alcohol self-administration regardless of dose (*p* > 0.05). However, LY2444296 administration significantly decreased alcohol self-administration at the 3 mg/kg (*t*_14_ = 2.24, *p* < 0.05) and 10 mg/kg (*t*_14_ = 2.68, *p* < 0.05) doses in alcohol-dependent rats (Fig. 4B). No differences in somatic withdrawal signs or active lever presses were observed, regardless of LY2444296 dose or history of alcohol dependence, at 2 or 4 weeks of abstinence (*p* > 0.05; Fig. 4A,B). No differences in inactive lever responses were observed, regardless of the rats’ histories of alcohol dependence or LY2444296 dose (*p* > 0.05; Fig. 4B).

## Discussion

The present study tested the ability of the KOP antagonist LY2444296 to decrease alcohol self-administration at different abstinence time points: 8 h, 2 weeks, and 4 weeks. This study also assessed whether LY2444296 administration also results in a parallel reduction of somatic withdrawal signs in male and female rats. Consistent with the extant literature, alcohol-dependent rats exhibited an increase in alcohol intake during alcohol dependence that was induced by CIE vapor exposure^42–47^. Notably, LY2444296 significantly reduced somatic withdrawal signs and alcohol intake in alcohol-dependent male and female rats at the 8 h abstinence time point. These results support the involvement of the DYN/KOP system in maladaptive actions of alcohol consumption and negative affective states that are associated with acute alcohol withdrawal and are particularly observed in subjects that are dependent on alcohol^12,48^.

In the present study, alcohol-dependent male and female rats exhibited a significant increase in alcohol intake that was accompanied by greater somatic withdrawal signs and higher BALs after self-administration sessions during week 6 of CIE vapor exposure. These results are consistent with previous studies that reported that rats with a history of alcohol dependence exhibit an increase in alcohol intake and an increase in somatic and motivational signs of withdrawal that are characteristic of dependence, which are observable acutely (i.e., approximately 6–8 h of abstinence from alcohol vapor)^42–47,49^. The present findings add to a growing body of literature that has shown that intermittent exposure to alcohol vapor effectively elicits physical, affective, and neurobiological signs of dependence. Indeed, these neuroadaptations that ultimately compromise functional integrity of the brain, particularly areas that are involved in response inhibition and reward-related behavior, may contribute to the emergence of negative emotional states, which are characterized by negative reinforcement, whereby dependent subjects seek relief from negative symptoms that are exacerbated during alcohol withdrawal, further perpetuating uncontrolled alcohol seeking and taking^50,51^.

The present study found that 3 and 10 mg/kg of the short-acting KOP antagonist LY2444296 significantly and selectively decreased alcohol self-administration in male and female rats with a history of alcohol dependence at 8 h of abstinence, without affecting alcohol self-administration in nondependent rats. These results are consistent with previous reports that manipulations of the DYN/KOP system via pharmacological blockade influence alcohol consumption in rodents^12^. Specifically, systemic LY2444296 administration before each drinking session prevented the stress-induced increase in alcohol consumption in male C57BL/6J mice that had undergone alcohol-dependence induction via CIE vapor exposure^32^. Furthermore, systemic administration of the long-acting KOP antagonist norBNI 24 h before a self-administration session that occurred at acute abstinence (6 h) effectively attenuated the escalation of alcohol intake that is commonly induced by chronic vapor exposure in dependent but not nondependent male Wistar rats^33,52^. Consistent with these findings, the systemic administration of CERC-501 selectively and dose-dependently decreased alcohol intake that was exacerbated following long-term intermittent access to 20% alcohol in male Wistar rats^35^. The present findings, together with the extant literature, provide further evidence that the DYN/KOP system is a prominent player in the loss of control over alcohol consumption under conditions of alcohol dependence. Indeed, given the selective effect of LY2444296 to decrease alcohol self-administration in dependent rats only, a possible explanation is that the DYN/KOP system becomes dysregulated as a function of alcohol exposure.

The observable withdrawal syndrome that consistently emerges in both animals and humans as a function of abruptly terminating chronic exposure to alcohol is one of the most prominent hallmarks of alcohol dependence and may reflect a hyperexcitability state of the central nervous system^53,54^. The most widely accepted working theory contends that alcohol dependence reflects an allostatic state that is furthered by the continuous dysregulation of neurophysiological systems, particularly those that are involved in reward and stress and are pushed beyond a normal homeostatic set point^55^. This allostasis leads to an increase in alcohol withdrawal symptoms and underlies the motivation for continued alcohol drinking^56^. Interestingly, the present study found that 3 and 10 mg/kg LY2444296 significantly decreased somatic withdrawal signs in alcohol-dependent male and female rats at 8 h of abstinence, an effect that, in turn, could be responsible for the significant decrease in alcohol consumption.

It is important to note, however, that these data are somewhat contrasting to previous work that showed blockade of KOP (by intra-CeA administration of norBNI) reduced alcohol consumption in dependent rats without any effect on alcohol withdrawal^57^. While the reason of this discrepancy is not clear, a simple explanation for the differential effects measured in the present study could be the different experimental approaches (e.g., route of administration, time of drug administration and testing, males *vs.* males and females). Moreover, it may also be relevant to consider norBNI’s complex pharmacokinetic and pharmacodynamic properties, which makes the interpretability of those results complicated, thus, those findings may not overlie the ones obtained with a short-acting KOP antagonist such as LY2444296. Indeed, the divergent effects of different KOP antagonists observed on the same behavioral endpoints may underlie distinctive downstream target systems^58^. This will need to be further investigated.

One of the most interesting findings of the present study was that LY2444296 administration decreased alcohol self-administration at 8 h of abstinence in alcohol-dependent rats, only. This was unsurprising, given that a recurring theme in the continuously expanding DYN/KOP literature is that the pharmacological blockade of KOPs is more effective in subjects with higher motivation for alcohol seeking (i.e., alcohol preference or dependence induction). The extant literature, like the present study, shows that KOP antagonist administration has no effect on basal, non-escalated alcohol consumption but attenuates escalated alcohol consumption in post-dependent subjects^32,33,35,52^. Furthermore, in alcohol-preferring (P) rats, which have long been thought to be a valid animal model of alcohol dependence, the administration of CERC-501 (i.e., LY2456302) reduced alcohol intake under free-access conditions^34^. One possible explanation for this selectivity is that the unique contribution of DYN/KOP transmission to motivational aspects of alcohol drinking does not play a significant role until anti-reward systems are sufficiently engaged or recruited. Consequently, in animals that are highly motivated to consume alcohol (i.e., alcohol-dependent rats), KOP transmission is potentially compromised and promotes the incentive to drink alcohol through negative reinforcement mechanisms, setting the stage for pharmacological inhibition of this system to be a potentially valid and effective therapeutic approach for individuals with AUD.

Interestingly, LY2444296 did not exert effects on either alcohol self-administration or somatic withdrawal signs at 2 or 4 weeks of abstinence in either nondependent or dependent rats in the present study. Previous research showed that dependent rats drank alcohol for up to 8 weeks following chronic exposure to alcohol via CIE vapor exposure^59^. To date, however, remaining unclear is how long the influence of the DYN/KOP system on the higher motivation for alcohol consumption following dependence remains into more protracted abstinence. Most studies that examined the role of DYN/KOP transmission on alcohol dependence reported effects of KOP antagonists during acute (between 6 and 12 h) withdrawal^33,35,52^. Nevertheless, one study found that norBNI administration in the CeA decreased alcohol intake after a protracted period of abstinence (~ 30 days), and this effect may be mediated, at least partially, by affective and not physical symptoms of withdrawal^57^. Thus, the present findings of somatic withdrawal signs at 2 and 4 weeks of abstinence are unsurprising because physical signs of withdrawal are thought to be short-lived upon abstinence. Indeed, even in humans, these somatic signs of withdrawal do not persist longer than an average of 7–10 days^60^. Altogether, these results may suggest that distinct neural substrates may underlie physical *vs*. affective aspects of the alcohol withdrawal syndrome, and their influence likely shifts, depending on the duration of abstinence.

One may argue that a limitation of the current study is that the specificity of the effect of LY2444296 on alcohol self-administration was not assessed because it was not tested against the self-administration of an alternative reinforcer such as a palatable sweet solution. However, previous studies have shown that other KOP antagonists (JDTic and norBNI, specifically) decreased alcohol, but not sucrose self-administration in rats^61^. Of note, it was shown that acute systemic administration of LY2444296 (at 1 and 3 mg/kg) had no effect on depressive- and anxiety-like behaviors of drug naïve single-housed rats^39^, and that norBNI administration, but not LY2444296, resulted in increased anxiolytic-like behavior in drug naïve mice^62^. Administration of LY2444296 failed to produce place aversion or preference and had no effect on locomotion or plasma corticosterone levels of cocaine naïve subjects^39^. Importantly, LY2444296 in the present study selectively reduced responses on the active, alcohol paired-lever, but not inactive lever, which suggests an effect that is specific to drug-directed behavior. In addition, the lack of effect of LY2444296 on alcohol self-administration in the nondependent group of rats further suggests a selectivity of this KOP antagonist to reduce alcohol self-administration following alcohol dependence. Nevertheless, future studies that examine the specificity of LY2444296 to alcohol self-administration versus the self-administration of a palatable conventional reinforcer are warranted. Furthermore, based on our experimental approach, it is difficult to ascertain that higher doses of LY2444296 would not decrease self-administration in either dependent or nondependent subjects. While pharmacological studies have found that LY2444296 shows good KOP selectivity even at doses as high as 30 mg/kg^38^, special attention should be paid to potential off target effects of LY2444296 at higher doses.

Overall, the present findings demonstrate that administration of the reversible KOP antagonist LY2444296 reduced physical signs of withdrawal and selectively decreased alcohol self-administration in rats with a history of alcohol dependence at 8 h abstinence. Nonetheless, caution is warranted when interpreting the ability of KOP antagonists to reduce drinking, and potentially relapse, in clinical populations. Though effective, some KOP antagonists that have been tested preclinically (e.g., norBNI, JTDic, and CERC-501) have shown unusual pharmacokinetic and pharmacodynamic properties, long-lasting effects, and poor solubility, thus hampering their clinical development. LY2444296 has shown to be a selective, short-acting, and centrally penetrating ligand that may prove to be both safe and effective in the clinical setting. Altogether, the present results highlight the importance of the DYN/KOP system in dependence-induced uncontrolled alcohol consumption and the relevance of preclinically evaluating KOP antagonists as a potential therapeutic avenue for AUD.

## Methods

### Animals

A total of 22 male and 22 female Wistar rats were purchased from Charles River Laboratories (Hollister, CA, USA). The rats weighed 150–170 g upon arrival in the laboratory. They were housed two per cage in a humidity- and temperature-controlled vivarium on a reverse 12 h/12 h light/dark cycle (lights OFF at 8:00 AM, lights ON at 8:00 PM) with access to food and water ad libitum. Before any experimental procedures, the rats were handled daily for 1 week to acclimate them to the housing and experimental conditions. All behavioral procedures were conducted during the dark cycle (i.e., between 8:00 AM and 8:00 PM). All animal procedures were conducted in strict adherence to the National Institutes of Health *Guide for the Care and Use of Laboratory Animals*^63^ and they were approved by the Institutional Animal Care and Use Committee of The Scripps Research Institute. Additionally, all methods and results were reported in accordance with *Animal Research: Reporting *In Vivo* Experiments (ARRIVE) Guidelines*^64,65^.

### Drugs

LY2444296 (Eli Lilly, Indianapolis, IN, USA) was diluted in distilled water with the addition of 10% lactic acid. Once homogenized, to maximize bioavailability of the compound, LY2444296 was administered orally (p.o.) at doses of 0, 3, and 10 mg/kg in a volume of 1 ml/kg. The 0 mg LY2444296 dose consisted of only the vehicle that was used to dissolve the compound (distilled water + 10% lactic acid).

### Alcohol self-administration training

Self-administration training was conducted as previously reported^43,46,47,66^. Importantly, no saccharin or sucrose fading procedure was required to induce voluntary alcohol intake in male and female rats. One week after the housing acclimation period and for the remainder of the training procedure (Fig. 1A), the rats were given access to alcohol in standard operant conditioning chambers (29 cm × 24 cm × 19.5 cm; Med Associates, St. Albans, VT, USA) during daily 30-min self-administration sessions (for 3 weeks; Fig. 1A) on a fixed-ratio 1 (FR1) schedule of reinforcement. During these sessions, responses on the right lever resulted in the delivery of 0.1 ml of 10% (w/v) alcohol (prepared in tap water from 95% w/v alcohol) in a drinking reservoir located in the center of the chamber’s front panel and the brief (0.5 s) illumination of a cue light above the lever. Responses on the left inactive lever were recorded but had no programmed consequences. To calculate total intake (g/kg), the total number of rewards (i.e., when responses on the active lever resulted in the delivery of alcohol) was normalized to the animal’s body weight on the day of that session. To assess whether the animals consumed the entirety of the self-administered alcohol, the drinking reservoirs were checked and confirmed to be dry after each self-administration session.

### Chronic intermittent ethanol vapor exposure

Once the 21 self-administration sessions were completed, half the rats (*n* = 22) were made alcohol-dependent via CIE vapor exposure, and the other half were exposed to air only (*n* = 22; nondependent group). During dependence induction (6 weeks; Fig. 1B), the rats underwent cycles of 14 h of alcohol vapor ON and 10 h of alcohol vapor OFF daily. Blood alcohol levels were measured using a gas chromatography-headspace blood analyzer (Agilent Technologies, Santa Clara, CA, USA). These BALs ranged between 150 and 250 mg%. For 3 weeks, all rats, regardless of dependence condition, remained undisturbed apart from measuring BALs during the last 30 min of vapor exposure (on Thursday) and scoring somatic signs of withdrawal (at 8 h of abstinence) once weekly (on Wednesday; Fig. 1B). Behavioral signs of withdrawal were measured by a laboratory technician who was blind to the experimental conditions using a scale that was adapted from Macey et al.^53^. Baseline withdrawal scores were measured before the last training session (see Fig. 1A). These withdrawal signs included measures of ventromedial limb retraction, vocalization (i.e., irritability to touch), tail stiffness, abnormal gait, and body tremors. Each of these behaviors was assigned a score of 0–2, based on severity: 0 = no signs, 1 = moderate, and 2 = severe. To confirm dependence and assess withdrawal severity, the sum of the five scores (0–10) was used as a quantitative measure. This approach was used because this model of alcohol dependence leads to motivational and somatic signs of withdrawal^45^. Baseline self-administration levels were calculated by averaging the last three self-administration training sessions (Fig. 1A). At weeks 4, 5, and 6 of alcohol vapor exposure (Fig. 1B), the animals underwent 30-min FR1 alcohol self-administration sessions 8 h after the alcohol vapor was turned OFF, when blood and brain alcohol levels are negligible, three times weekly (Monday, Wednesday, and Friday). To further corroborate alcohol dependence, withdrawal signs and BALs were measured after the self-administration sessions at week 6 of CIE vapor exposure. Importantly, air-exposed rats (nondependent) were subjected to the same BAL assessments, withdrawal testing, and alcohol self-administration sessions as the dependent subjects during weeks 4–6.

### Effects of LY2444296 on alcohol self-administration at acute (8 h) abstinence

During week 7 and week 8 of CIE vapor exposure (Fig. 1C), effects of LY2444296 (0, 3, and 10 mg/kg) on alcohol self-administration at acute (8 h) abstinence were tested. LY2444296 was administered orally (p.o.) 30 min before the start of the self-administration sessions at an acute abstinence time point (8 h after the alcohol vapor was turned OFF). To control for possible order effects of LY2444296 dosing on self-administration, each rat was tested with all doses of LY2444296 in random order using a Latin-square design every other session. On days between testing, the rats underwent regular self-administration sessions without LY2444296 administration (Fig. 1C).

### Effects of LY2444296 on somatic withdrawal signs and alcohol self-administration at acute, late, and protracted abstinence

A separate group of male and female rats (*n* = 8 females, *n* = 8 males) that were prepared in parallel were used to test LY2444296’s ability to reduce somatic withdrawal signs and alcohol intake at acute (8 h), late (2 weeks), and protracted (4 weeks) abstinence (see Fig. 1D). During weeks 7 and 8 of CIE vapor exposure, at 8 h of abstinence, the rats received LY2444296 orally (p.o.) at doses of 0, 3, and 10 mg/kg; 30 min later, they were scored for behavioral signs of withdrawal (as previously described) and then placed in the operant chambers for a regular self-administration session. Once acute abstinence testing was completed (end of week 8), the rats were removed from the vapor inhalation chambers and started a 4-week abstinence period (Fig. 1D). At 2 weeks of abstinence, 30 min before being scored for somatic withdrawal signs and being placed in the operant chambers for a self-administration session, half the rats that were tested at 8 h abstinence received either 0 or 10 mg/kg LY2444296. Following the 2-week abstinence test, the rats were returned to the vivarium to undergo an additional 2 weeks of abstinence (Fig. 1D), corresponding to an overall 4-week abstinence period (the same rats that were tested at 8 h and 2 weeks abstinence were used). At this time point, the rats that received 0 mg/kg LY2444296 at 2 weeks of abstinence received 10 mg/kg LY2444296 and the rats that received 10 mg/kg LY2444296 at 2 weeks of abstinence received 0 mg/kg LY2444296 30 min before scoring somatic withdrawal signs and placing them in the operant chambers for a self-administration session.

### Statistical analyses

A mixed two-way ANOVA was used to analyze the acquisition of alcohol self-administration during the 3 weeks of training, with session and lever (i.e., active *vs*. inactive) as within- and between-subjects factors, respectively. Alcohol intake during CIE vapor exposure (i.e., baseline vs. week 6) was analyzed using a mixed two-way ANOVA, with time and alcohol dependence as independent factors. Time and alcohol dependence were also factors in a mixed two-way ANOVA with withdrawal scores (log10-transformed for the statistical analysis, back-transformed for graphical representations). Pearson correlation coefficients were calculated to establish the linear relationship between responses of the active lever and BALs at week 6 of CIE. The effect of LY2444296 on active lever presses during self-administration at acute abstinence (8 h) was analyzed using a mixed two-way ANOVA, with alcohol dependence and treatment (i.e., 0 vs. 3 and 10 mg/kg LY2444296) as sources of variance. Given the results obtained in the original first experiment testing the effects of LY2444296 on alcohol self-administration at 8 h abstinence, planned comparisons using two-tailed Student’s *t*-tests were used to examine hypotheses that LY2444296 would affect alcohol self-administration at 8 h, as well as withdrawal scores at 8 h, 2 weeks, and 4 weeks of abstinence in dependent animals. Significant interactions and main effects in the ANOVAs were followed by Bonferroni’s and Tukey’s (where appropriate) multiple-comparison post hoc tests. The data are expressed as the mean + SEM. Values of *p* < 0.05 were considered statistically significant. The statistical analyses were performed using Prism 8 software (GraphPad, San Diego, CA, USA).

## Data Availability

The datasets generated during and/or analyzed during the present study are available from the corresponding author upon reasonable request.
